# Upregulated SSB Is Involved in Hepatocellular Carcinoma Progression and Metastasis through the Epithelial-Mesenchymal Transition, Antiapoptosis, and Altered ROS Level Pathway

**DOI:** 10.1155/2023/5207431

**Published:** 2023-02-04

**Authors:** Hao Wu, Zhixin Zhang, Xinyu Han, Sai Zhang, Jinrui Zhang, Pinsheng Han, Youcheng Zhang, Yi Bai, Yamin Zhang

**Affiliations:** ^1^The First Central Clinical School, Tianjin Medical University, Tianjin 300070, China; ^2^Department of Genetics, School of Basic Medical, Tianjin Medical University, Tianjin 300070, China; ^3^School of Medicine, Nankai University, Tianjin 300192, China; ^4^Department of Hepatobiliary Surgery, Tianjin First Central Hospital, School of Medicine, Nankai University, Tianjin 300192, China

## Abstract

Hepatocellular carcinoma (HCC) is one of the most common malignant tumors with high morbidity and mortality. Therefore, finding new diagnostic and therapeutic targets is vital for HCC patients. Recent studies have shown that dysregulation of RNA-binding proteins is often associated with cancer progression. Several studies have reported that the RNA-binding protein SSB can promote cancer occurrence and progression and is linked to tumor epithelial-mesenchymal transition (EMT), which could be a new diagnostic marker and therapeutic target. However, the expression and function of SSB in HCC remain to be elucidated. Therefore, this study is aimed at clarifying the expression and biological function of SSB in HCC through bioinformatics analysis combined with in vitro experiments. We found that SSB is highly expressed in HCC and is associated with the poor prognosis of HCC patients, and it can serve as an independent unfavorable prognostic factor. Knockdown of SSB can inhibit the growth of HCC cells in vitro, increase the level of apoptosis and the expression of pro-apoptosis-related proteins, and decrease the expression of antiapoptotic proteins. Meanwhile, SSB knockdown reduced HCC cell invasiveness, and the expression of EMT-related proteins changed significantly. We also found that the gene SSB was associated with the level of oxidative stress in liver cancer cells, and the level of intracellular reactive oxygen species (ROS) increased after knockdown of SSB. The results of bioinformatics analysis also showed that high expression of SSB may affect the effect of checkpoint blockade (ICB) therapy. In conclusion, we found that SSB is highly expressed in HCC and that upregulated SSB can promote the proliferation and metastasis of HCC through antiapoptotic, altered intracellular oxidative stress level, and EMT pathways, which can serve as a new diagnostic marker and therapeutic target, and patients with high SSB expression may not have obvious ICB therapy effect.

## 1. Introduction

According to the most recent statistics, more than 8 million new hepatocellular carcinoma (HCC) cases are diagnosed yearly, causing more than 4 million deaths [1]. The incidence of HCC is highest in Asia, especially in China, which accounts for almost half of the global cases [2]. While several therapies have been developed in recent years, including liver transplantation, hepatectomy, targeted therapy, ablation, and transcatheter arterial chemoembolization, the survival rate in HCC patients is only 18% for five years [3]. This is mainly because the early clinical manifestations of HCC patients are not apparent, and most treatments are limited to early-stage patients. Thus, exploring novel therapeutic targets based on molecular biomarkers is urgently needed for HCC patients, which is crucial for the survival of HCC patients.

SSB, also known as RNA-binding protein La or La-related protein 3 (LARP3), belongs to the LA-related protein family and is an RNA-binding protein mainly expressed in the nucleus [4, 5]. RNA-binding proteins (RBPs) participate in posttranscriptional regulation, such as splicing, polyadenylation, and stabilization [6]. Abnormal posttranscriptional regulatory processes may lead to tumorigenesis, and the mechanisms underlying this have been elucidated, including genomic changes and posttranscriptional modifications [6, 7]. RBPs also influence oncogenes and tumor suppressor genes' expression and function [8]. Studies have shown that different members of LARP family are involved in the occurrence and progression of cancer [9]. The SSB is highly expressed in various tumors, including chronic myelogenous leukemia, ovarian cancer, and head and neck cancer [10–12]. Aberrant expression of SSB proteins contributes to increased cancer cell proliferation, migration, invasion, and chemoresistance and promotes tumor growth in mice [12–14]. In addition, the SSB protein has RNA chaperone activity that promotes the processing of noncoding precursor RNAs but also stimulates the translation of selective mRNAs that encode cancer-promoting and antiapoptotic genes [15]. Oxidative stress plays an important role in the occurrence and development of cancer, and it also affects the prognosis of patients [16]. Thus, SSB may also affect the level of oxidative stress in tumor cells. However, there are still few reports and in-depth studies on the role of SSB in HCC. Therefore, the specific molecular mechanism of SSB in HCC needs to be explored urgently.

This study is aimed at investigating the prognostic role and cancer-promoting molecular mechanisms of SSB in HCC to identify SSB as a potential therapeutic target for HCC patients. Through bioinformatics analysis combined with in vitro experiments, we confirmed for the first time that SSB was significantly upregulated in HCC tissues and correlated with poor prognosis. It could promote the progression and metastasis of HCC through epithelial-mesenchymal transition (EMT), altered intracellular oxidative stress level, and antiapoptotic pathways and may affect the efficacy of immunotherapy.

## 2. Materials and Methods

### 2.1. Data Acquisition

The transcriptome sequencing and clinicopathological data were retrieved from TCGA-LIHC dataset (https://www.portal.gdc.cancer.gov/), and GTEx data (https://gtexportal.org/) was also downloaded. In addition, the GSE121248 dataset was downloaded from Gene Expression Omnibus (GEO, https://www.ncbi.nlm.nih.gov/geo/) for external expression validation. The differential expression of SSB protein in normal liver and HCC tissue was analyzed using immunohistochemistry (IHC) data from the Human Protein Atlas database (HPA, https://www.proteinatlas.org/). In addition, we performed SSB pancancer expression and survival analysis using TIMER 2.0 (http://timer.cistrome.org/) and GEPIA (http://gepia.cancer-pku.cn/) databases.

### 2.2. Independent Prognostic and Clinical Characteristic Analysis of Gene SSB

The patients were divided into high- and low-expression groups according to the median expression level of gene SSB. Survival analysis was performed using Kaplan-Meier and log-rank tests. We also assessed the relationship between SSB and clinical characteristics such as age, sex, stage, grade, and TNM stage. In addition, univariate and multivariate Cox analyses were performed for SSB and clinical characteristics to determine independent prognostic indicators. Analyses were performed using R software's “survival” and “survminer” packages (v 4.0.2). *P* < 0.05 was considered significant.

### 2.3. Gene Set Enrichment Analyses (GSEA)

SSB expression was classified into high- and low-expression phenotypes based on the median value of gene SSB expression. Enrichment analysis was performed with default parameter settings. The random number set is 1000 times. The FDR < 0.05 are significantly enriched in GSEA.

### 2.4. Cell Culture

The human normal liver cell line (L-02) and hepatocarcinoma cell lines (HepG2 and SMMC-7721) were purchased from ICell Bioscience Inc (Shanghai, China) and the Cell Bank of the Chinese Academy of Sciences (Shanghai, China). The L-02 and SMMC-7721 cell lines were cultured in RPMI-1640 medium (Gibco, Grand Island, USA), and HepG2 cell line was cultured in DMEM medium (Gibco, Grand Island, USA) and all media containing 10% fetal bovine serum (FBS, Gibco, Grand Island, USA) and 1% penicillin/streptomycin (Beyotime, Shanghai, China). All cells were preserved in a humidified incubator (37°C, 5% CO_2_).

### 2.5. Transfection of Hepatocarcinoma Cell Lines

Tianjin Medical University's Basic Experiment Center extracted and synthesized the pLKO.1-SSB knockdown and pLKO.1-SSB (scramble) plasmid. Three different SSB short hairpin RNAs (shRNAs) were designed and transfected into hepatoma cells using lentiviral packaging plasmids. The specific information is as follows: sh-scramble (5′-TGTGAGGAACTTGAGATCT-3′), sh-SSB1 (5′-CCTGCATCCAAACAACAGAAA-3′) and sh-SSB2 (5′-GCTGAAATGAAATCTCTAGAA-3′). The shSSB base sequence is based on previously studied and verified sequence information [17].

### 2.6. RNA Extraction and Quantitative Real-Time Polymerase Reaction (qRT-PCR) Assay

A total RNA extraction kit (Solarbio, Beijing, China) was used to extract total RNA from cells following the manufacturer's instructions. Afterward, the RNA was quantified using the One-Step SYBR Prime Script RT-PCR kit (Takara, Japan). The expression of SSB was normalized to GAPDH and was analyzed using the 2^−△△CT^ method [18]. The sequence information of the SSB primers is in Supplementary Table 1.

### 2.7. Cell Viability Assay

Cell viability was measured by cell counting kit-8 (CCK-8, Boster, Wuhan, China) assay. Following transfection, each group's cells were seeded into 96-well plates (1 × 10^4^ cells/well) and cultured in the incubator for 24, 48, and 72 h. After that, 10 *μ*L of CCK-8 was added to each well and placed in the incubator (37°C, 5% CO_2_) for 1 h. Then, the absorbance at 450 nm was measured using a microplate reader (EnSpire, USA).

### 2.8. Colony Formation Assay

Cells in each group were digested with 0.25% trypsin, seeded into 6-well plates, and cultured in a medium containing 10% FBS for two weeks. When visible colonies were formed, cells were fixed in 20% methanol for 10–15 min, then stained with 0.1% crystal violet for 10 min at room temperature, and finally, the colonies in each well were counted [19].

### 2.9. Wound Healing Assay

Cells from each group were seeded into 6-well plates for 24 h before replacing the medium with a serum-free medium. The cells were scratched with a yellow pipette tip and cultured for 48 h. The cells were photographed at 0, 6, 24, and 48 h. Image-J (V7.0) software was used to quantify the distance between two edges of the wound surface at each time point.

### 2.10. Apoptosis Detection by Flow Cytometry

Cell apoptosis was detected using the Annexin-V-FITC/PI Apoptosis Detection Kit (Solarbio, Beijing, China). After digesting the cells of each group, 500 *μ*L of binding buffer, 5 *μ*L of FITC-Annexin V, and 5 *μ*L of PI working solution were added. Then, the cells were incubated at room temperature in the dark for 15 min. Finally, the level of apoptosis was detected by flow cytometry (BD Accuri C6 Plus, USA) [20]. Data were processed using FlowJo (V10.0) software.

### 2.11. Cell Cycle Detection by Flow Cytometry

Cell cycle distribution was assessed using the Cell Cycle Detection Kit (KeyGEN, Nanjing, China). Cells in each group were first fixed with 70% ethanol at 4°C overnight, then collected by centrifugation, mixed with 500 *μ*L of RNase A/PI working solution, and incubated at room temperature for 30 min. Finally, flow cytometry was used for detection. Data were processed using ModFit LT (V3.1) software.

### 2.12. Measurement of ROS Levels by Flow Cytometry

The ROS levels were measured using the ROS assay kit (Solarbio, Beijing, China). Briefly, cells were resuspended in a serum-free medium containing 10 mM DCFH-DA. Subsequently, flow cytometry was performed to detect the DCF fluorescence intensity. Finally, the mean fluorescence intensity (MFI) was calculated.

### 2.13. Transwell Assay

Cell invasion assays were performed using a transwell cell culture chamber coated with Matrigel (Corning Costar, Inc.) according to the manufacturer's instructions. First, the cells of each group were inoculated in a serum-free medium in the upper chamber, and the medium containing 20% FBS was filled in the lower chamber. After that, cells were cultured in an incubator (37°C, 5% CO_2_) for 24 h. Transwell migration experiments are similar to the above steps, except that Matrigel is not added to the migration experiments. Data were processed using ImageJ (V7.0) software.

### 2.14. Western Blotting

Total protein was extracted from cells in each group using RIPA lysate (Solarbio, Beijing, China). The protein concentration was measured by the BCA (KeyGEN, Nanjing, China) method. Then, the proteins were separated by SDS-PAGE (Boster, Wuhan, China). After the target proteins were transferred to the nitrocellulose filter (NC, Boster, Wuhan, China) membrane, it was blocked with 5% skimmed milk at room temperature for 1 h. Next, these membranes were incubated with primary antibodies such as SSB (1 : 1000, Boster, Wuhan, China), E-cadherin (1 : 1000, Cell Signaling Technology, USA), N-cadherin (1 : 1000, Cell Signaling Technology, USA), ZO-1 (1 : 3000, Proteintech, Wuhan, China), Vimentin (1 : 1000, ImmunoWay, USA), GAPDH (1 : 20000, Affinity Biosciences, China), Caspase3 (1 : 1000, Santa Cruz, USA), BCL2 (1 : 1000, Abcam, USA), BCLXL(1 : 1000, Abcam, USA), and BAX (1 : 2000, Abcam, USA) overnight at 4°C. Subsequently, these membranes were incubated with the corresponding secondary antibody for 1 h at room temperature. The membranes were exposed using an imaging system (Bio-Rad, Hercules, USA) and quantified using ImageJ (V7.0) software.

### 2.15. Estimation of Correlations between SSB Expression Level and Immune Cells

Spearman's correlation analysis was used to describe the correlation between the SSB and various immune cells. The correlation analyses between SSB expression and immune cells were performed using the R package “ggstatsplot” (v 4.0.2). *P* < 0.05 was considered significant.

### 2.16. Prediction of Immune Checkpoint Blockade (ICB) Therapy Response

According to the median SSB expression, HCC samples were divided into two groups, and Tumor Immune Dysfunction and Exclusion (TIDE) algorithm was used to predict the potential response of ICB in the two groups [21].

### 2.17. Evaluation of the Relationship between SSB Expression and Immune Checkpoint-Related Genes

SIGLEC15, TIGIT, CD274, HAVCR2, PDCD1, CTLA4, LAG3, and PDCD1LG2 are genes related to immune checkpoints. The expression values of these eight genes were extracted to clarify the relationship between immune checkpoint-related genes and SSB expression. The above results were statistically analyzed by R software (v 4.0.2). *P* < 0.05 was considered significant.

### 2.18. Statistical Analysis

The bioinformatics data analyses of this study were performed using R software (v 4.0.2). The Wilcoxon rank-sum test determined the comparison between the two groups. Analysis of in vitro experimental data was conducted using GraphPad Prism 8.0 statistical software. A one-way analysis of variance (ANOVA) compared the differences among three or more groups, and the Student *t*-test evaluates the significance between the two groups. *P* < 0.05 was considered to be statistically significant.

## 3. Results

### 3.1. SSB Is Upregulated in HCC and Associated with Poor Prognosis of HCC Patients

**Figure 1:**
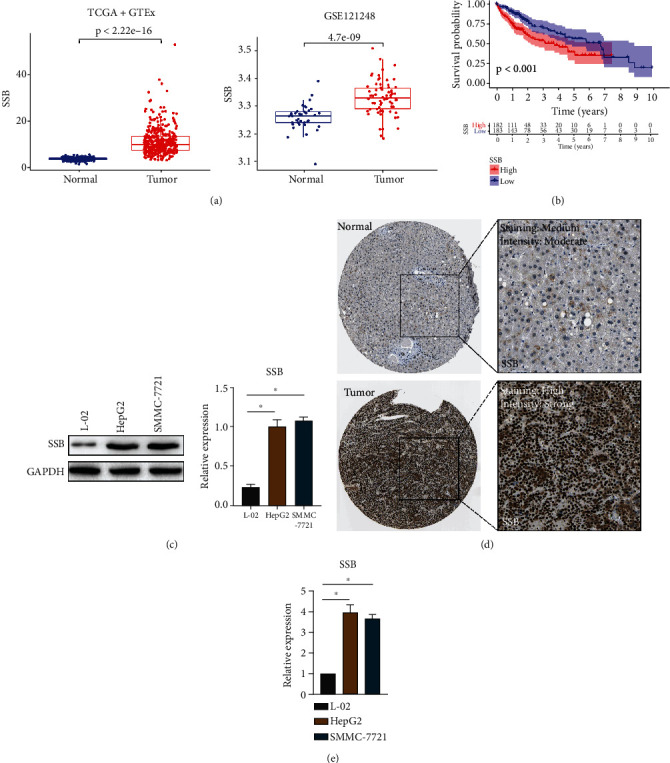
SSB is highly expressed in HCC tissues or cells. (a) SSB is highly expressed in HCC tissues compared to normal tissues based on TCGA and GEO databases. (b) High expression of SSB is associated with poor prognosis in HCC patients. (c) SSB is highly expressed in HepG2 and SMMC-7721 HCC cells compared with normal hepatocyte L-02. (d) SSB is highly expressed in HCC tissues compared to normal liver tissues based on the HPA IHC database. (e) qRT-PCR results showed that SSB expression was significantly upregulated in HepG2 and SMMC-7721 cells compared with L-02 cells. Data are shown as mean ± SD. ^∗^*P* < 0.05.

A total of 374 tumor samples (TCGA) and 276 normal samples (TCGA + GTEx) were obtained. In addition, 70 HCC and 37 normal samples were obtained from the GEO database (GSE121248). As displayed in Figure 1(a), in TCGA and GEO databases, SSB was highly expressed in HCC tissues compared with normal samples (*P* < 0.05). Survival analysis results suggested that patients with high SSB expression had a poor prognosis (Kaplan-Meier and log-rank test, *P* < 0.05, Figure 1(b)). In vitro experiments, western blot, and qRT-PCR showed that SSB was highly expressed in HCC cell lines HepG2 and SMMC-7721 compared with normal liver cell line L-02 (*P* < 0.05, Figures 1(c) and 1(e)). The IHC data of SSB in HCC tissues and normal tissues were downloaded from the HPA database. The results are depicted in Figure 1(d), SSB was highly expressed in HCC tissues compared with normal liver tissues.

### 3.2. Evaluation of the Correlation between SSB Gene Expression and Clinicopathological Characteristics

**Figure 2:**
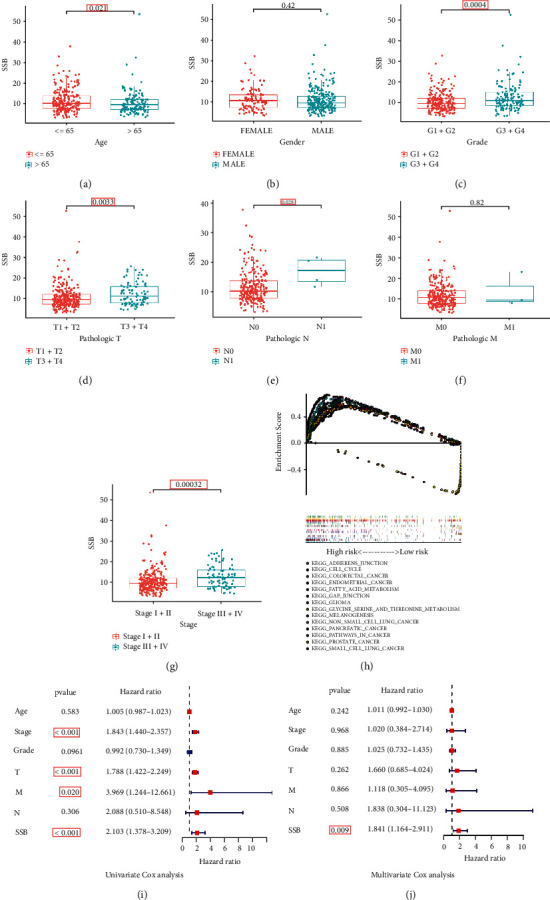
SSB is a prognostic risk factor involved in multiple cancer-related signaling pathways (a–g). The correlation of SSB expression with clinicopathological variables. (h) GSEA suggests that SSB is involved in multiple cancer-related pathways. (i, j) Univariate and multivariate Cox analyses confirmed SSB as an independent prognostic risk factor.

The clinical information of patients in TCGA-LIHC cohort is shown in Table 1. Increased SSB expression correlated with age (*P* = 0.021, Figure 2(a)), histological grade (G3/G4 vs. G1/G2, *P* = 0.0004, Figure 2(c)), T stage (T3/T4 vs. T1/T2, *P* = 0.0033, Figure 2(d)), N stage (*P* = 0.028, Figure 2(e)), and AJCC stage (III/IV vs. I/II, *P* = 0.00032, Figure 2(g)). However, there was no correlation between gender, M stage, and SSB expression (*P* > 0.05, Figures 2(b) and 2(f)).

### 3.3. SSB Is an Independent Prognostic Risk Factor and Is Involved in Multiple Cancer-Related Signaling Pathways

The potential KEGG pathway between high and low SSB expression was analyzed using GSEA method. The results are described in Figure 2(h). The high expression of SSB enriches a variety of cancer-related signaling pathways, such as “colorectal cancer,” “glioma,” “pancreatic cancer,” “small cell and non-small-cell lung cancer,” and other cancer signaling pathways. In addition, the high expression of SSB also affects the cell cycle and enriches signaling pathways related to cancer invasion and metastasis, such as “adherens junction” and “gap junction.” On the other hand, the low expression of SSB mainly enriches the metabolism-related signaling pathways.

Independent prognostic analyses of SSB and clinicopathological characteristics were assessed using univariate and multivariate Cox regression methods. Univariate Cox analysis showed that AJCC stage, T stage, M stage, and SSB expression were relevant to overall survival (all *P* < 0.05, Figure 2(i)), and the multivariate Cox analysis showed that only SSB expression was relevant to overall survival, which also indicated that SSB was an independent prognostic factor (*P* < 0.05, Figure 2(j)).

### 3.4. Pancancer Analysis of SSB

**Figure 3:**
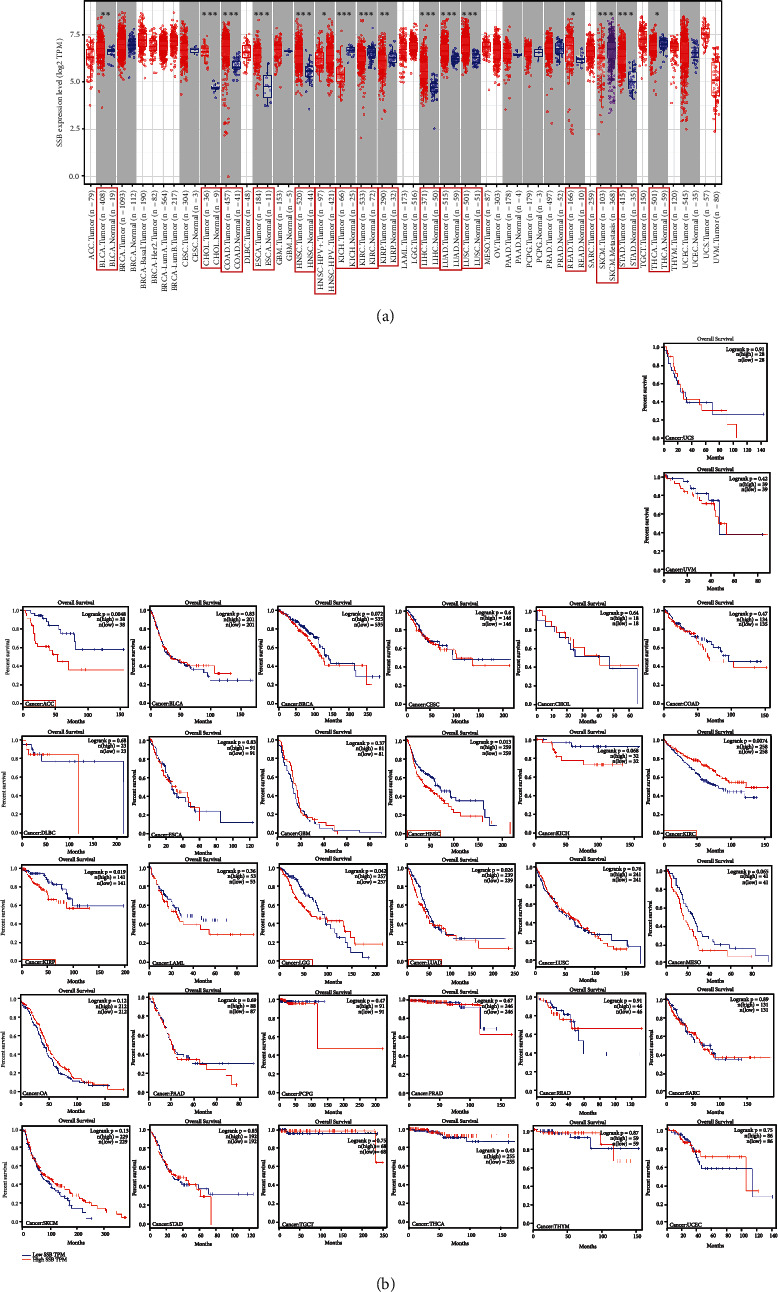
Expression and Kaplan-Meier survival analysis of SSB in pancancer. (a) SSB pancancer expression analysis. (b) Kaplan-Meier survival analysis of SSB in pancancer.

The pancancer expression analysis results are shown in Figure 3(a). SSB is differentially expressed in other 14 cancer types (*P* < 0.05). In 9 of the 14 types of cancer, SSB was highly expressed in tumor tissues compared to normal tissues, including urothelial bladder carcinoma (BLCA), cholangiocarcinoma (CHOL), colon adenocarcinoma (COAD), esophageal carcinoma (ESCA), head and neck squamous cell carcinoma (HNSC), lung adenocarcinoma (LUAD), lung squamous cell carcinoma (LUSC), rectum adenocarcinoma (READ), and stomach adenocarcinoma (STAD). In the remaining five cancer types, SSB was lowly expressed in tumor tissues compared with normal tissues or metastasis tissues, including kidney chromophobe (KICH), kidney renal clear cell carcinoma (KIRC), kidney renal papillary cell carcinoma (KIRP), skin cutaneous melanoma (SKCM), and thyroid carcinoma (THCA).

According to the median value of SSB gene expression, patients were divided into low- and high-expression groups, and survival analysis was performed using Kaplan-Meier and log-rank tests. The results of the SSB pancancer survival analysis are illustrated in Figure 3(b). SSB has survival significance in 6 cancer types (*P* < 0.05). The high expression of SSB in adrenocortical carcinoma (ACC), HNSC, KIRP, brain lower grade glioma (LGG), and LUAD has a poor prognosis, while high SSB expression in KIRC has a good prognosis.

### 3.5. SSB Knockdown Inhibited the Proliferation and Migration Ability of HepG2 and SMMC-7721 Cell Lines and Increased Intracellular ROS Levels

**Figure 4:**
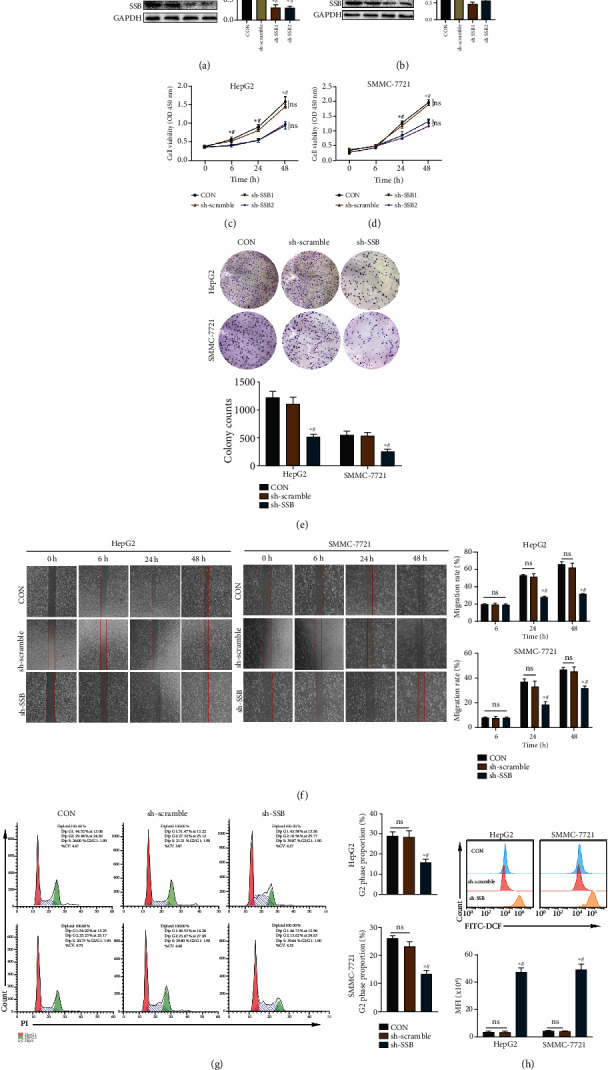
SSB promoted HepG2 and SMMC-7721 cell proliferation in vitro. (a, b) The knockdown efficiency of SSB in hepatoma cell lines HepG2 and SMMC-7721. (c, d) The cell viability of hepatoma cell lines SMMC-7721 and HepG2 decreased after SSB knockdown. (e) Cell clone formation assay showed that the proliferation of hepatoma cells decreased after the knockdown of SSB. (f) Cell scratch assay showed that hepatoma cell migration ability decreased after the knockdown of SSB. (g) The flow cytometry cell cycle results showed that the proportion of cells in the proliferative phase decreased after the knockdown of SSB. (h) The levels of ROS in hepatocellular carcinoma cell lines increased after SSB knockdown. Data are shown as mean ± SD. ^∗^ refers to a statistically significant difference in the sh-SSB group compared to the CON group, *P* < 0.05. # refers to the statistically significant difference in the sh-SSB group compared to the sh-scramble group, *P* < 0.05. NS: no statistical difference.

Three types of shRNA (sh-scramble, sh-SSB1, and sh-SSB2) were designed to transfect HepG2 and SMMC-7721 cell lines. The purpose of sh-SSB1 and sh-SSB2 is to knock down the gene SSB. After determining the knockdown efficiency, we performed the CCK-8 experiment. The results are shown in Figures 4(a) and 4(b). In HepG2 and SMMC-7721 cell lines, SSB expression was significantly reduced in both sh-SSB1 and sh-SSB2 groups (*P* < 0.05) compared to CON and sh-scramble groups, demonstrating that both sh-SSB1 and sh-SSB2 have good knockdown efficiency. The subsequent CCK8 experiments showed that in HepG2 and SMMC-7721 cell lines, knockdown of SSB could significantly inhibit the proliferation of HCC cell lines compared with CON and sh-scramble groups (*P* < 0.05, Figures 4(c) and 4(d)). However, there was no significant difference in cell viability between CON and sh-scramble groups (*P* > 0.05).

We only selected sh-SSB1 as sh-SSB to knock down SSB for subsequent in vitro experiments. The results of clone formation experiments showed that in HepG2 and SMMC-7721 cell lines, the number of cell clones formed after knockdown of SSB was significantly lower than that in CON and sh-scramble group cells (*P* < 0.05, Figure 4(e)). The results of cell scratch experiments are shown in Figure 4(f). In HepG2 and SMMC-7721 cell lines, there was no significant difference in cell migration rates between groups at 6 hours (*P* > 0.05), while at 24 and 48 hours, the cell migration rate of the sh-SSB group was significantly lower than that of the CON and sh-scramble groups (*P* < 0.05). The 24-hour transwell migration assay confirmed that the cell migration ability of the knockdown SSB group decreased (*P* < 0.05, Supplementary Figure 1). In addition, we used flow cytometry to detect the cell cycle of the cells in each group. The results showed that in the HepG2 and SMMC-7721 cell lines, the proportion of cells in the proliferative G2 phase in the sh-SSB group decreased compared with that in the CON and sh-scramble groups (*P* < 0.05, Figure 4(g)).

We used flow cytometry to detect ROS levels, and the results showed that the intracellular ROS levels were increased in hepatoma cells in the SSB knockdown group compared with the CON and sh-scramble groups (*P* < 0.05, Figure 4(h)).

### 3.6. Knockdown of SSB Promotes Apoptosis in HepG2 and SMMC-7721 Cell Lines

**Figure 5:**
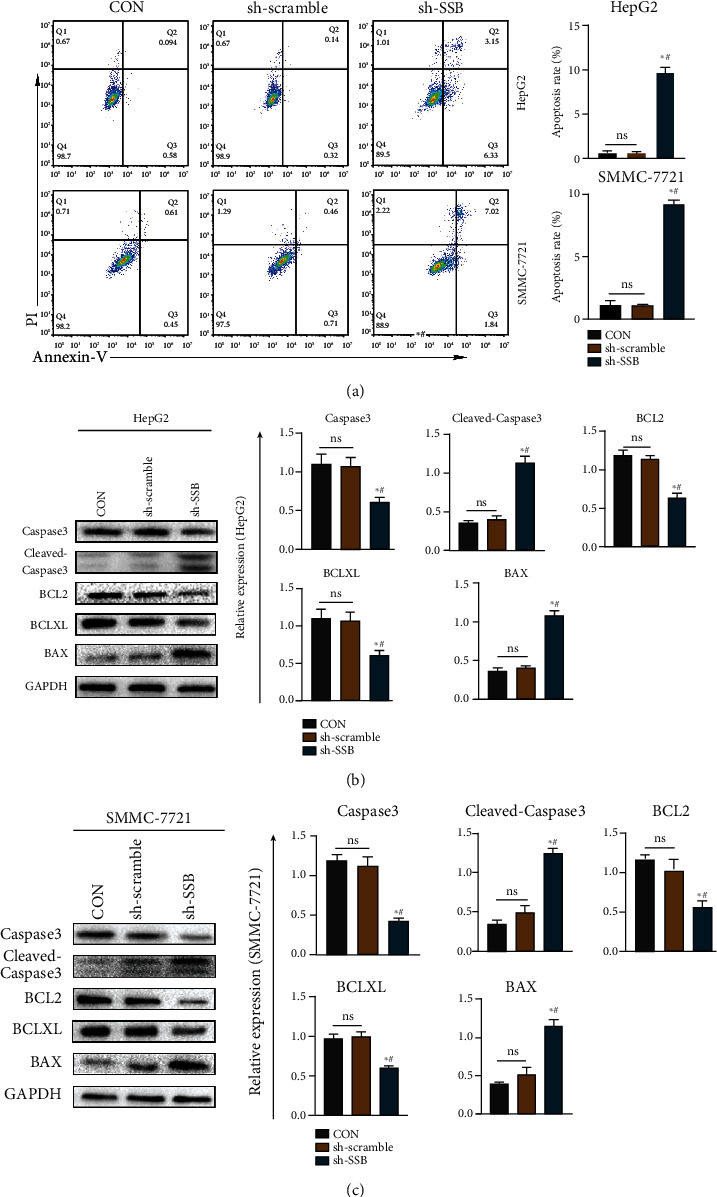
Knockdown of SSB can promote the apoptosis of HCC cells. (a) After the knockdown of SSB, the level of apoptosis in hepatoma cell lines HepG2 and SMMC-7721 increased. (b) Expression of apoptosis-related proteins in hepatoma cell line HepG2. (c) Expression of apoptosis-related proteins in hepatoma cell line SMMC-7721. Data are shown as mean ± SD. ^∗^ refers to a statistically significant difference in the sh-SSB group compared to the CON group, *P* < 0.05. # refers to the statistically significant difference in the sh-SSB group compared to the sh-scramble group, *P* < 0.05. NS: no statistical difference.

After collecting the cells of each group, the apoptosis level was detected by flow cytometry. The results are shown in Figure 5(a). In the HepG2 and SMMC-7721 cell lines, the apoptosis rate was increased in the sh-SSB group compared with the CON and sh-scramble groups (all *P* < 0.05). However, there was no significant difference in apoptosis rate between the CON group and the sh-scramble group (*P* > 0.05). In addition, we extracted the total protein of each group of cells for western blot experiments. The results showed that in HepG2 and SMMC-7721 cell lines, knockdown of SSB significantly increased the expression of cleaved-Caspase3 and BAX proapoptotic protein and decreased the antiapoptotic protein BCL2 and BCLXL expression compared with the CON group and sh-scramble group (*P* < 0.05, Figures 5(b) and 5(c)).

### 3.7. SSB Is Involved in the Invasion and Metastasis of HCC through the EMT Pathway

**Figure 6:**
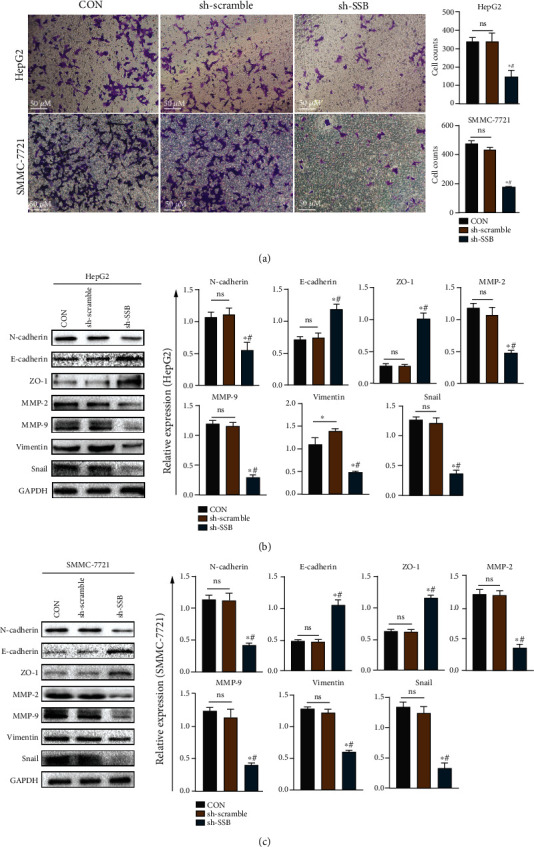
SSB can promote the invasion of hepatoma cells through the EMT pathway. (a) Cell invasion assay of HepG2 and SMMC-7721 cells. (b) EMT-related protein expression in the hepatoma cell line HepG2. (c) Expression of EMT-related proteins in hepatoma cell line SMMC-7721. Data are shown as mean ± SD. ^∗^ refers to a statistically significant difference in the sh-SSB group compared to the CON group, *P* < 0.05. # refers to the statistically significant difference in the sh-SSB group compared to the sh-scramble group, *P* < 0.05. NS: no statistical difference.

Transwell invasion assay was used to detect the effect of knockdown of SSB on the invasion function of HepG2 and SMMC-7721 HCC cell lines. The results are depicted in Figure 6(a). In HepG2 and SMMC-7721 cells, the invasive ability of the cells after knockdown of SSB decreased compared with CON and sh-scramble groups (*P* < 0.05). In addition, we performed western blot analysis to clarify the expression of EMT-related proteins. The results are displayed in Figures 6(b) and 6(c). In HepG2 and SMMC-7721 cells, after the knockdown of SSB, the expression of N-cadherin, MMP-2, MMP-9, Vimentin, and Snail proteins was decreased compared with CON and sh-scramble groups, and the expression of E-cadherin and ZO-1 proteins was increased (*P* < 0.05).

### 3.8. High Expression of SSB May Affect the Effect of Immunotherapy

**Figure 7:**
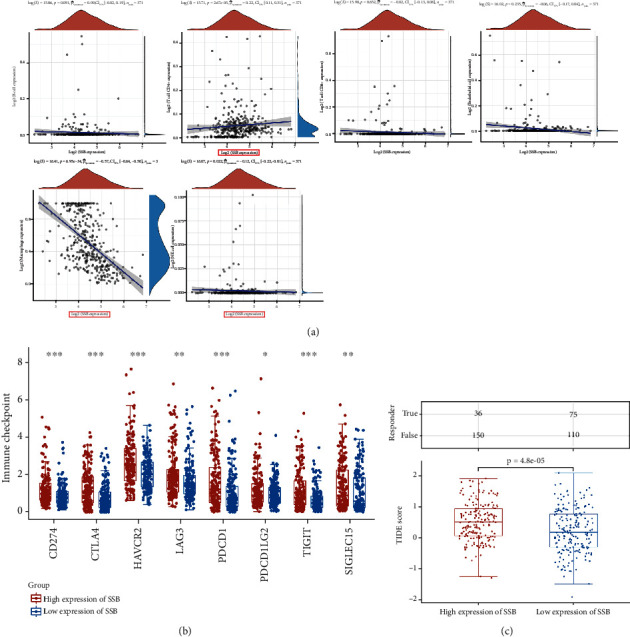
High expression of SSB may affect the effect of ICB therapy. (a) Spearman correlation analysis between SSB and immune cells. (b) Expression analysis of genes related to immune checkpoints and SSB. (c) Statistical table of immune response of samples in different groups in the prediction results and the TIDE scores in different groups in the prediction results. ^∗^*P* < 0.05, ^∗∗^*P* < 0.01, and ^∗∗∗^*P* < 0.001.

The expression of SSB was negatively correlated with macrophages and NK cells but significantly positively correlated with CD4^+^T cells (*P* < 0.05, Figure 7(a)). All eight immune checkpoint-related genes showed high expression levels in patients with high SSB expression (*P* < 0.05, Figure 7(b)). In TCGA-LIHC cohort, the high-expression SSB group had significantly higher TIDE scores than the low-expression group (*P* < 0.05, Figure 7(c)). As judged by the TIDE score, patients with high expression of SSB may be insensitive to ICB treatment. In other words, patients with low expression of SSB may be more sensitive to ICB treatment.

## 4. Discussion

HCC remains a disease with a poor prognosis and high mortality due to its high late diagnosis rate, metastasis rate, and rapid malignant progression [22]. However, early diagnosis and effective treatment measures can significantly improve the survival rate of HCC patients. Therefore, it is vital to find new diagnostic markers and therapeutic targets for the early progression of HCC [23, 24]. In recent years, due to the rapid development of omics technology, we have gained a deeper understanding of the pathogenesis, diagnosis, and treatment of HCC [25].

In this study, we first analyzed the transcriptome data of HCC samples in TCGA database, concluding that SSB is highly expressed in HCC tissues and is an independent prognostic risk factor for HCC patients. Afterward, we verified the expression of SSB using GEO and HPA IHC databases. Furthermore, we used western blot and qRT-PCR assays to confirm the high expression of SSB in HCC. SSB expression and clinicopathological characteristics of HCC patients were analyzed by univariate and multivariate Cox analysis, suggesting that SSB was an independent prognostic risk factor. In addition, GSEA suggested that the high expression of SSB was associated with different tumor-related signaling pathways. The pancancer research project was initiated by TCGA in 2012, and this research relies on multiomics high-throughput database information mining to find the similarities and differences among tumors and provide guidance for the subsequent diagnosis, prognosis, and other treatment plans of tumors [26]. We subsequently performed pancancer expression and survival analysis of SSB, and the results showed that SSB was upregulated in multiple cancers and affected patient prognosis, which was also consistent with previous studies [10–12]. Although the incidence of different cancers and the depth of research are inconsistent, through pancancer analysis, the mechanism, and effective drugs can be compared in-depth, and the same target can be used for different cancers. In this study, we mainly studied the prognostic significance and related mechanisms of SSB in patients with liver cancer and proved that the high expression of SSB is not conducive to the prognosis of patients. From the perspective of HCC treatment, specific antibodies can be designed to try to block the function of target molecules such as SSB, to achieve the possibility of treatment and prevention of migration and recurrence. Now, the development of tumor therapeutic drugs has crossed the boundaries of tumor tissue types, and the development of specific drugs for the target molecules of pancancer analysis can treat a variety of cancers of different tissue origins.

To further explore the biological function of SSB in HCC cells, we knocked down SSB in two HCC cell lines (HepG2 and SMMC-7721). The results showed that after the knockdown of SSB, the proliferation ability of HepG2 and SMMC-7721 HCC cell lines decreased, and the cell cycle results also showed that the proportion of cells in the proliferative phase decreased. Apoptosis is a form of programmed cell death that plays a crucial role in homeostasis, infection, injury, and clearance of senescent cells [27]. After the knockdown of SSB, the level of apoptosis increased, the expression levels of anti-apoptotic-related proteins decreased, and the expression levels of pro-apoptotic-related proteins increased in HCC cells. This also indicates that the gene SSB may promote the proliferation of HCC cells through the antiapoptotic pathway.

Oxidative stress is associated with many physiological and pathological processes [28, 29]. Disruption of cellular oxidative stress homeostasis has been shown to be associated with the development of HCC [30, 31]. Related studies have shown that ROS play multiple roles in cancer [16, 32, 33]. On the one hand, ROS is crucial for cancer cell survival and tumor growth, and on the other hand, excess ROS can lead to cancer cell death [34]. Importantly, tumor cells utilize cellular antioxidant systems to counteract the prodeath effects of ROS. There is increasing evidence that proteins with antioxidant activity are involved in tumorigenesis and metastasis [35]. By conducting a series of experiments, we verified that modulation of SSB affects ROS levels and may thereby lead to the proliferation and metastasis of HCC.

Epithelial-mesenchymal transition (EMT) is the biological process of transforming epithelial cells into cells with a mesenchymal phenotype through a specific program. It plays a vital role in embryonic development, chronic inflammation, tissue remodeling, cancer metastasis, and various fibrotic diseases [36–38]. In addition, studies have shown that the malignant progression of many cancer types is likely to depend entirely on the activation of EMT in tumor cells [39–41]. After EMT activation, E-cadherin and ZO-1 were inhibited, resulting in loss of epithelial cell morphology. The cells change to a spindle-shaped mesenchymal morphology and express markers associated with the mesenchymal cell state, such as N-cadherin, Vimentin, and fibronectin [42, 43]. Previous studies have shown that the gene SSB is associated with EMT, and the motility and invasion ability of squamous cell carcinoma cells are decreased after knockdown of SSB, and the expression of matrix metalloproteinase 2 (MMP-2) protein is significantly decreased [12, 17].

Tumor invasion and metastasis are a complex, continuous process involving multiple molecules, especially matrix metalloproteinases (MMPs) [44]. Degradation of basement membrane and extracellular matrix (ECM) by MMPs promotes tumor cell invasion and proliferation [45]. MMP-2 and MMP-9, among all MMP members, have been linked to tumor metastasis [46, 47]. Therefore, the invasive ability of tumor cells should be proportional to the expression of MMP-2 and MMP-9. Snail is an essential inducer of EMT and can strongly inhibit the expression of E-cadherin [48]. In addition, studies have revealed that Snail expression levels are elevated in metastatic lesions of ovarian cancer [49]. In the present study, we confirmed by transwell invasion assay that the invasive ability of HepG2 and SMMC-7721 cell lines was decreased after SSB knockdown. Afterward, a western blot was performed to detect the expression of EMT marker proteins. The results showed that after the knockdown of SSB, the expression of epithelial marker protein N-cadherin decreased, E-cadherin and other mesenchymal proteins increased, and MMP-2 and MMP-9 expression also decreased. This is also consistent with the results of previous studies. All the above results indicate that SSB can participate in the invasion and progression of HCC through the EMT pathway.

ICB therapy has revolutionized the treatment of cancer in humans, and it can significantly improve patient outcomes by reversing the immunosuppressive microenvironment by reducing the likelihood of tumor immune escape [50, 51]. TIDE uses a panel of gene expression signatures to assess two distinct tumor immune escape mechanisms, including tumor-infiltrating cytotoxic T lymphocyte (CTL) dysfunction and CTL rejection by immunosuppressive factors. The higher the TIDE prediction score, the higher the possibility of immune evasion, indicating that patients are less likely to benefit from ICB treatment [21]. To further evaluate the potential immune mechanism of SSB in LIHC, we analyzed the level of SSB-related immune infiltration. The results showed that the level of SSB expression was positively correlated with the infiltration level of CD4+ T cells in LIHC. In addition, the expression of SSB was negatively correlated with macrophages and NK cells. We investigated the association of SSB with immune checkpoints, including SIGLEC15, TIGIT, CTLA4, CD274, HAVCR2, LAG3, PDCD1, and PDCD1LG2, which are associated with ICB responses. PD-1, CTLA4, LAG3, and HAVCR2 are T cell depletion markers [52], and the T cell depletion is a major factor contributing to immune dysfunction in cancer patients. In this study, all these marker genes were positively correlated with SSB expression in LIHC. Since high expression of immune checkpoints is associated with T cell exhaustion and poor prognosis, this also partially explains the cancer-promoting role of SSB. The TIDE score of the high-expressing SSB group was higher, which also suggested that it is not suitable for ICB treatment in the HCC patients with high SSB expression.

In conclusion, we confirmed that SSB is highly expressed in HCC tissues, which is a prooncogene and may be involved in the proliferation, invasion, and metastasis of HCC through antiapoptosis, changing the level of cellular oxidative stress, and EMT pathway. We have demonstrated for the first time that the gene SSB can affect the level of cellular oxidative stress and it can be used as a new target for the diagnosis and treatment of HCC patients. However, HCC patients with high SSB expression may be insensitive to ICB therapy.

## Figures and Tables

**Table 1:** The clinical characteristics in TCGA-LIHC cohort.

Parameter	Type	Patients
Status	Alive	241 (64.96%)
	Dead	130 (35.04%)

Age	Mean (SD)	59.4 (13.5)
	Median [Min, Max]	61 [16, 90]

Gender	Female	121 (32.61%)
	Male	250 (67.39%)

Histologic grade	G1	55 (14.82%)
	G2	178 (47.98%)
	G3	120 (32.35%)
	G4	13 (3.50%)
	Unknown	5 (1.35%)

Pathologic M	M0	269 (72.51%)
	M1	3 (0.81%)
	Unknown	99 (26.68%)

Pathologic N	N0	253 (68.19%)
	N1	4 (1.08%)
	Unknown	114 (30.73%)

Pathologic T	T1	184 (49.60%)
	T2	92 (24.80%)
	T3	79 (21.29%)
	T4	13 (3.50%)
	Unknown	3 (0.81%)

AJCC stage	Stage I	174 (46.90%)
	Stage II	85 (22.91%)
	Stage III	84 (22.58%)
	Stage IV	4 (1.08%)
	Unknown	24 (6.53%)

## Data Availability

The databases were downloaded from TCGA database (https://www.portal.gdc.cancer.gov), GEO database (https://www.ncbi.nlm.nih.gov/geo/), TIMER2.0 (http://timer.cistrome.org/), GEPIA (http://gepia.cancer-pku.cn/index.html), and HPA (https://www.proteinatlas.org/). Further inquiries can be directed to the corresponding author.

## Supplementary Materials

Supplementary Materials Supplementary Table 1: the sequence information of the SSB primers. Supplementary Figure 1: hepatoma cell transwell migration assay.
