# Supplementary material for: Upregulated SSB Is Involved in Hepatocellular Carcinoma Progression and Metastasis through the Epithelial-Mesenchymal Transition, Antiapoptosis, and Altered ROS Level Pathway
Source: Oxid Med Cell Longev. 2023 Feb 4;2023:5207431. doi: 10.1155/2023/5207431 (PMC9922187; doi:10.1155/2023/5207431)
Supplement: Supplementary Materials — Supplementary Table 1: the sequence information of the SSB primers. Supplementary Figure 1: hepatoma cell transwell migration assay. [file 5207431.f1.docx]

Supplementary Table 1: The sequence information of the SSB primers.

| Gene | Forward | Reverse |
| --- | --- | --- |
| SSB | 5’-GCCTTCATCGCTCACATCAG-3’ | 5’-CTTGTCCCGTGGCAAATTGA-3’ |
| GAPDH | 5ʹ-GGAGCGAGATCCCTCCAAAAT-3ʹ | 5ʹ-GGCTGTTGTCATACTTCTCATGG-3ʹ |

Supplementary Figure 1: Hepatoma cell transwell migration assay.


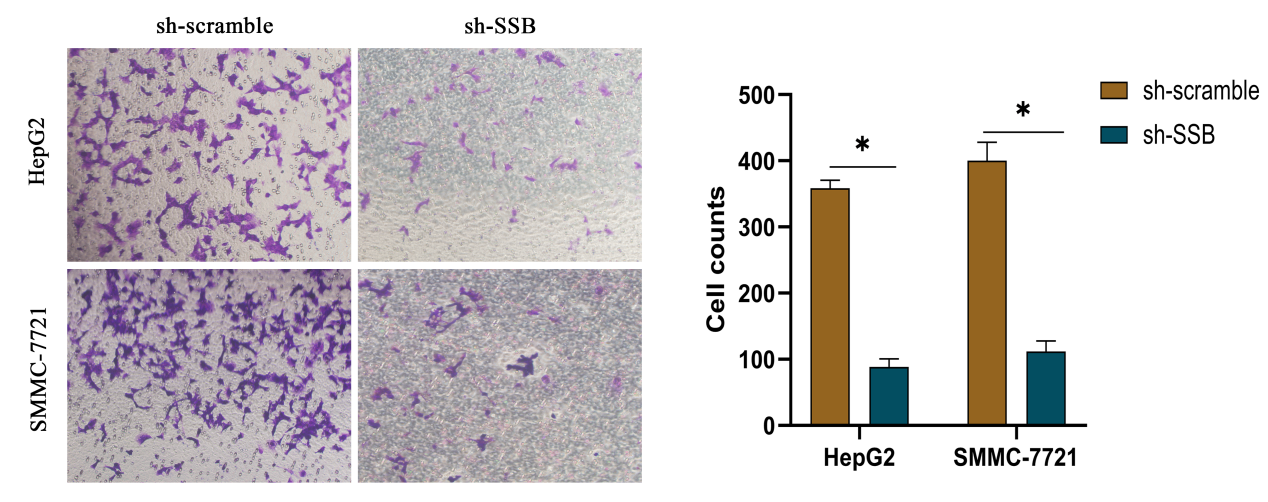


Supplementary Figure 1.Transwell migration assay showed that hepatoma cell migration ability decreased after the knockdown of SSB. Data are shown as mean ± SD. **P* < 0.05
